# Gait patterns in Prader-Willi and Down syndrome patients

**DOI:** 10.1186/1743-0003-7-28

**Published:** 2010-06-21

**Authors:** Veronica Cimolin, Manuela Galli, Graziano Grugni, Luca Vismara, Giorgio Albertini, Chiara Rigoldi, Paolo Capodaglio

**Affiliations:** 1 Bioeng. Dept., Politecnico di Milano, p.zza Leonardo Da Vinci 32, 20133, Milano, Italy; 2 IRCCS "San Raffaele Pisana", Tosinvest Sanità, Roma, Italy; 3 Divisione di Auxologia, Ospedale San Giuseppe, Istituto Auxologico Italiano, Via Cadorna 90, I-28824, Piancavallo (VB), Italy; 4 Laboratorio di Ricerca in Biomeccanica e Riabilitazione, Ospedale San Giuseppe, Istituto Auxologico Italiano, Via Cadorna 90, I-28824, Piancavallo (VB), Italy

## Abstract

**Background:**

Prader-Willi (PWS) and Down Syndrome (DS) are two genetic disorders characterised by some common clinical and functional features. A quantitative description and comparison of their patterns would contribute to a deeper understanding of the determinants of motor disability in these two syndromes. The aim of this study was to measure gait pattern in PWS and DS in order to provide data for developing evidence-based deficit-specific or common rehabilitation strategies.

**Methods:**

19 PWS patients (17.7-40 yr) and 21 DS patients (18-39 yr) were evaluated with an optoelectronic system and force platforms for measuring kinematic and kinetic parameters during walking. The results were compared with those obtained in a group of normal-weight controls (Control Group: CG; 33.4 + 9.6 yr).

**Results and Discussion:**

The results show that PWS and DS are characterised by different gait strategies. Spatio-temporal parameters indicated a cautious, abnormal gait in both groups, but DS walked with a less stable strategy than PWS. As for kinematics, DS showed a significantly reduced hip and knee flexion, especially at initial contact and ankle range of motion than PWS. DS were characterised by lower ranges of motion (p < 0.05) in all joints than CG and PWS. As for ankle kinetics, both PWS and DS showed a significantly lower push-off during terminal stance than CG, with DS yielding the lowest values. Stiffness at hip and ankle level was increased in DS. PWS showed hip stiffness values close to normal. At ankle level, stiffness was significantly decreased in both groups.

**Conclusions:**

Our data show that DS walk with a less physiological gait pattern than PWS. Based on our results, PWS and DS patients need targeted rehabilitation and exercise prescription. Common to both groups is the aim to improve hypotonia, muscle strength and motor control during gait. In DS, improving pelvis and hip range of motion should represent a major specific goal to optimize gait pattern.

## Background

Prader-Willi (PWS) and Down Syndrome (DS) are two different chromosomal disorders characterised by some common clinical features, such as obesity, muscular hypotonia, ligament laxity and mental retardation.

PWS is a complex multisystemic disorder equally affecting males and females. The genetic basis is the absent expression of the paternally active genes in the PWS critical region on chromosome 15 [1]. It is characterized by muscular hypotonia, ligament laxity, hyperphagia, severe obesity, short stature, hypogonadism, mental retardation and dysmorphic features. Both hypotonia and excessive body weight may affect the development of motor and functional skills of PWS individuals [2,3].

DS is caused by trisomy of chromosome 21 (Hsa21) and is associated with a number of signs and symptoms including learning disabilities, heart defects, craniofacial dysmorphia and childhood leukaemia [4]. Physical activity patterns of DS are influenced by ligaments' laxity and reduced muscle strength and tone [5]. Similarly to PWS, the DS-related obesity may contribute to the reduced motor skills observed in this population [6,7].

Among the latter, gait disorders are common in both syndromes. They tend to progressively worsen as the clinical picture advances, severely limiting the patients' quality of life.

In previous studies, gait analysis has mainly focused on DS with special reference to their specific associated orthopaedic conditions and biomechanical limitations.

Caselli et al. [8] reported that walking in children and adolescents with DS was characterized by a ''Chaplinesque'' pattern with external rotation of the hips, increased knee flexion and valgus and external rotation of the tibia. Roizen et al [9] observed a plano-valgus foot with marked pronation in DS children impairing postural stability and gait. In adolescents and adults with DS, the same authors described hallux valgus, "hammer toe" deformities, plantar fasciitis and early onset of foot arthritis associated with severe flat feet, with an overall negative impact on ambulation and function. Parker et al. [10]) studied the gait pattern of six DS children using video analysis and reported a poor heel-toe rocking during the stance phase and increased abduction of the lower limb to facilitate foot clearance. Galli et al. [11] observed a prolonged hip flexion during the gait cycle, an increased knee flexion in the sagittal plane at the initial contact and reduced ankle plantar-flexion ability at toe-off in 63 DS children. Their gait was further characterized by a significant decrease in plantar-flexor moments and generated ankle power. More recently [12], the same authors demonstrated that DS patients yield stiffer hips and less stiff ankles as compared to normal-weight counterparts.

To our knowledge, only one quantitative study has investigated the biomechanical strategy during gait in PWS [13]. These authors compared the gait patterns of adult PWS patients with those obtained in obese and normal-weight individuals. Their results showed that PWS walked slower, with shorter stride length, lower cadence and longer stance phase compared with both obese and controls. Similarly, their ranges of motion at knee and ankle level as well as their plantar-flexor activity were significantly reduced.

Despite a different aetiology, the two genetic conditions do share several clinical and functional features. Whether the biomechanical determinants of such motor limitations are the same is still unknown and needs further investigations at various levels. Rehabilitation specialists are challenged by motor disability in PWS and DS patients, but they fail to provide evidence-based treatment modalities. A deeper understanding of the causes of their gait abnormalities, and ultimately of their motor disability, may well generate novel spin-offs for rehabilitation planning and treatment. 3-D gait analysis (GA) is nowadays the most accurate tool to investigate the gait pattern. From a clinical perspective, measuring the joint angular displacement, reactions, moments and powers provides insight into the 'how' (kinematics) and the 'why' (kinetics) of the movement observed. No studies up to now have addressed this issue of defining quantitative differences in gait strategy between DS and PWS. We could hypothesise that due to their common clinical and functional features rehabilitation strategies aimed at reducing motor disability in these two genetic conditions may share some common bases. In this wake of evidence, appropriate and effective rehabilitation and exercise prescription could be tailored to the unveiled specific or common deficits.

The aim of our study was therefore to identify, quantify and compare the spatiotemporal, kinematic and kinetic parameters of gait in PWS and DS adult patient using 3D-Gait analysis (GA) and compare their results with those obtained in a group of normal-weight control subjects.

## Methods

### Participants

Nineteen PWS and twenty-one DS patients matched for age, height, weight and body mass index (kg/m2: BMI), were enrolled in this study (Table 1).

**Table 1:** Clinical characteristics of the study groups.

	PWS patients	DS patients	Control Group
Participants (M/F)	19 (11/8)	21 (12/9)	20 (10/10)
Age (years)	25.7 ± 6.1	25.7 ± 6.1	33.4 ± 9.6
Height (cm)	153.1 ± 6.90*	149.2 ± 9.10*	173.3 ± 5.01
Weight (Kg)	97.5 ± 19.0*	84.5 ± 10.9*	66.9 ± 8.5
BMI (Kg/m^2^)	41.3 ± 6.0*	37.2 ± 5.8*	22.8 ± 3.2
*All values are mesn ± sd			

Data are expressed as mean (standard deviation).

+ = p < 0.05, PWS GROUP versus DS GROUP; *= p < 0.05 compared with Control Group.

The PWS patients had been periodically hospitalised at the Ospedale San Giuseppe, Istituto Auxologico Italiano, Piancavallo (VB), Italy. At admission, they underwent a clinical assessment and attended a 4-week comprehensive rehabilitation program. All patients showed the typical PWS clinical phenotype [14]. Cytogenetic analysis was performed in all participants; 13 out of them had interstitial deletion of the proximal long arm of chromosome 15 (del15q11-q13). Uniparental maternal disomy for chromosome 15 (UPD15) was found in 6 individuals.

The DS patients were all referred to the IRCCS "San Raffaele Pisana", Tosinvest Sanità, Roma, Italy. The distribution of chromosomal anomalies is pure trysomy 21 in all of the DS patients.

All PWS and DS patients were able to understand and complete the test and walk independently without aids.

Twenty age-matched individuals were included as controls (Control Group: CG). Exclusion criteria for the control group included prior history of cardiovascular, neurological or musculoskeletal disorders. They showed normal flexibility and muscle strength and no obvious gait abnormalities.

The study was approved by the Ethics Committees of the two Institutes for PSW and DS patients. Written informed consent was obtained by the parents or, when applicable, by the patients.

## Methods

The complete evaluation consisted of: clinical examination, video recording and 3 D Gait Analysis (GA).

The PWS patients were evaluated at the Movement Analysis Lab of the San Giuseppe Hospital, Istituto Auxologico Italiano, Piancavallo (VB), Italy, using an optoelectronic system with 6 cameras (460 VICON, Oxford Metrics Ltd., Oxford, UK) with a sampling rate of 100 Hz, and two force platforms (Kistler, CH).

DS patients were assessed at the Movement Analysis Lab of the IRCCS "San Raffaele Pisana", Tosinvest Sanità, Roma, Italy, using a 12-camera optoelectronic system (ELITE2002, BTS, Milan, Italy) with a sampling rate of 100 Hz, two force platforms (Kistler, CH) and 2 TV camera Video system (BTS, Italy) synchronized with the system and the platforms for videorecording.

To evaluate the kinematics of each body segment, passive markers were positioned on the participants' body, as described by Davis [15]

After placement of the markers, subjects were asked to walk barefoot at their own natural pace (self-selected speed) along a walkway containing the force platforms at the mid-point. Kinematic and kinetic data were collected for each patient from five trials in order to guarantee reproducibility of the results.

### Data comparability between Laboratories

A potential bias of this study is the variability of data originating from the two different laboratory settings. Variability can indeed be present if different systems for kinematic acquisition are used and differences in marker positioning are evident. Therefore, two control subjects were tested in both laboratories in order to assess the consistency of the data measured with the two systems, the markers' placement and the data collection procedures.

### Data analysis

All graphs obtained from GA were normalized as % of gait cycle and kinetic data were normalized for individual body weight.

For each participant (both patients and controls), three out of five trials, consistent in terms of gait pattern (spatio-temporal, kinematic and kinetic were considered for analysis.

Using specific software (BTS EliteClinic, version 3.4.109, for the Movement Analysis Lab of IRCCS "San Raffaele Pisana", Tosinvest Sanità, and Polygon Application, version 2.4, for the Movement Analysis Lab of San Giuseppe Hospital, Istituto Auxologico Italiano, data were exported in .txt and .xls files. From these data format we identified and calculated some parameters (time/distance parameters, angles joint values in specific gait cycle instant, peak values in joint power graphs) using the STATISTICA computer package (StatSoft Inc., Tulsa, OK, USA). This procedure was performed by the same operator to ensure data reproducibility. The following parameters were evaluated:

Spatio-temporal parameters:

- % stance (as % of the gait cycle);

- mean velocity, normalised to the individual's height (1/s);

- anterior step length, normalised to individual's height;

- cadence: number of steps in a time unit (steps/min).

Kinematics:

- the mean value (Mean PT index) of pelvis on sagittal plane during the gait cycle;

- the values of angle of ankle (AIC index), knee (KIC index) and hip joint (HIC index) at the Initial Contact (IC);

- the values of maximal ankle dorsiflexion during stance phase (AMSt index) and the maximal flexion of the knee (KMSw index) during swing phase;

- the values of minimal ankle dorsiflexion in stance phase (AmSt index), knee (KmSt index), and hip flexion (HmSt index) during the gait cycle;

- the range of motion of the pelvis on the coronal (PO-ROM index) and transversal (PR-ROM index) plane; the range of motion of hip on coronal (HAA-ROM index) and sagittal (HFE-ROM) plane; the range of motion of knee (KFE-ROM index) on sagittal plane; the range of motion of ankle on sagittal plane during stance phase (ADP-ROM index).

Kinetics:

- the maximum ankle power during terminal stance (maximum value of positive ankle power; APMax index, W/Kg) and the same index normalized to the velocity of progression (APMax norm index, m/s^2^). This parameter represents the push-off capacity during walking and is related to the forward propulsive power during gait.

Joint Stiffness:

In order to evaluate the effect of ligament laxity and hypotonia on joint kinetics and kinematics, hip and ankle stiffness (hip stiffness: Kh index; ankle stiffness: Ka index) were expressed by plotting the values of the flexion-extension moment versus the flexion-extension angle over the gait cycle interval between 10% and 30%. The 10% to 30% interval (corresponding to the second rocker) of the gait cycle was selected and the linear regression was fitted. The angular coefficient of the linear regression corresponded to the joint stiffness index, as described in previous studies [16,17]. Knee stiffness was not included in this study due to the lack of linear relation between kinematics and kinetics.

These parameters were chosen in line with the studies on gait strategy in PWS [13] and DS [11,12].

### Statistical analysis

All the previously defined parameters were computed for each participant and then the mean values and standard deviations of all indexes were calculated for each group.

Data of the two individuals acquired in the two different laboratories were compared with the Wilcoxon's test, in order to detect significant differences due to marker placement and data collection procedures in the two laboratories. Data of the PWS and DS were compared using Mann-Whitney U tests, in order to detect significant differences between PWS and DS. The patients' and the controls' data were compared with Mann-Whitney U tests. Null hypotheses were rejected when probabilities were below 0.05.

## Results

### Data comparability between Laboratories

We verified that marker placement and data collection procedures in the two laboratories were compared and the differences of all the computed kinematic and kinetic data of the two healthy subjects were not statistically different (p > 0.05). On this basis data from other 18 control subjects who served as the CG were acquired at the Movement Analysis Lab of the San Giuseppe Hospital, Istituto Auxologico Italiano.

### Comparison between PWS and DS

In Table 1 the clinical characteristics of PWS, DS and CG are reported.

Age was not significantly different among groups. BMI, weight and height were similar in PWS and DS but significantly different from CG. In order to take in account the variability in height and weight between pathological groups and CG, stride length was normalised to the subject's height and kinetic data were normalised to the subject's weight.

In Tables 2 the mean values and standard deviations of the spatio-temporal and kinematic indices considered in this study for PWS, DS and CG are reported.

**Table 2:** Spatio-temporal and kinematic parameters of the study groups.

	PWS GROUP	DS GROUP	Control Group	P-value (PWS vs. DS)
*Spatio-temporal parameters*				
%stance (% gait cycle)	63.88 (9.12)* +	60.95 (3.10)	59.45 (1.45)	0.0034
Anterior step length	0.33 (0.04)*+	0.28 (0.04)*	0.88 (0.21)	0.0087
Cadence (step/min)	111.76 (9.12)+	94.32 (11.24)*	111.80 (4.80)	0.0001
Velocity (1/s)	0.63 (0.10)*+	0.45 (0.08)*	0.78 (0.06)	0.0002
*Pelvis (°)*				
Mean PT	20.86 (8.84)*	18.01 (4.84)*	6.53 (6.97)	0.0859
PO-ROM	8.46 (3.36)*+	6.05 (2.12)	6.01 (2.53)	0.0003
PR-ROM	10.95 (3.61)	9.95 (2.69)	10.72 (5.32)	0.2803
*Hip joint (°)*				
HIC	45.88 (12.82)* +	33.74 (11.46)*	27.23 (9.57)	0.0001
HmSt	1.52 (10.66)*	1.32 (9.20)*	-14.83 (9.60)	0.9242
HFE-ROM	45.37 (5.99)+	32.42 (8.61)*	43.52 (4.76)	0.0001
HAA-ROM	16.89 (3.95)*+	12.58 (4.21)	10.71 (3.06)	0.0001
*Knee joint (°)*				
KIC	8.42 (6.64)* +	2.54 (7.75)	4.06 (6.63)	0.0006
KmSt	-2.58 (5.92)	0.41 (8.09)	0.12 (3.82)	0.0538
KMSw	53.25 (7.61)* +	41.06 (10.68)*	59.01 (6.18)	0.0001
KFE-ROM	55.83 (8.05)*+	43.81 (11.34)*	60.28 (6.31)	0.0001
*Ankle joint (°)*				
AIC	-3.15 (9.24)*	-3.55 (4.88)*	1.81 (6.87)	0.4503
AMSt	14.80 (8.81)* +	11.47 (4.26)*	21.04 (5.16)	0.0303
AmSt	-10.25 (8.79)+	-2.94 (3.91)*	-8.74 (9.40)	0.0002
ADP-ROM	25.16 (3.38)+	14.41 (3.77)*	27.72 (6.56)	0.0001
AMSw	13.77 (9.03)* +	6.15 (4.40)	8.63 (9.93)	0.0003

Data are expressed as mean (standard deviation).

+= p < 0.05, PWS GROUP versus DS GROUP; *= p < 0.05 compared with Control Group.

(ROM: Range Of Motion; PT: Pelvic Tilt; PO: Pelvic Obliquity; HIC: Hip at IC; HFE: Hip Flex-Extension; HAA: Hip Ab-Adduction; KIC: Knee at IC; KFE: Knee Flex-Extension; AIC: Ankle at IC; ADP: Ankle Dorsi-Plantarflexion; IC: Initial Contact; St: Stance; Sw: Swing; M: maximum value; m: minimum value)

PWS patients were characterised by longer stance duration than DS and normal cadence when compared to DS patients. In terms of anterior step length and velocity of progression, both PWS and DS showed reduced values as compared to CG, with PSW performing better than DS.

As for the pelvic joint, PWS and DS showed a forward tilted pelvis in the sagittal plane (Mean PT index) with no significant differences between groups. Their pelvic range of motion in the transversal plane (PR-ROM index) was close to normal. In the frontal plane (PO-ROM index), PWS group was characterised by a higher pelvic range of motion during walking as compared to DS and CG.

The hip joint exhibited excessive flexion during the whole gait cycle (HIC and HmSt indices) in both PWS and DS patients, but PWS walked with a more flexed hip at the initial contact (HIC index). The hip range of motion (HFE-ROM index) was close to normal in PWS and reduced in DS. So despite an increased hip flexion in PWS, its range of motion was more functional as compared to DS.

As for hip ab-adduction, the PWS patients were characterised by an increased hip movement in the frontal plane as compared to DS (HAA-ROM index).

The knee flex-extension plot revealed that, whilst the PWS group showed an excessively flexed knee as compared to DS at the initial contact (KIC index), both PWS and DS obtained values close to normal in midstance (KmSt index). In the swing phase, the maximum value of knee flexion (KMSw index) was reduced in both PWS and DS, with generally higher mean values in PWS leading to a wider joint range of motion (KFE-ROM index) than that observed in DS.

The analysis of the ankle kinematics showed a plantar flexed position with reduced range of motion (ADP-ROM index) during the whole stance phase (AIC, AMSt indices) in DS, while PWS were limited at the initial contact (AIC index) and during midstance (AMSt index), even if their dorsiflexion capacity, and therefore their range of motion (ADP-ROM index), in stance phase was higher than that observed in DS. During the swing phase, PWS were characterised by higher values of ankle dorsiflexion when compared to DS and CG. All these differences are significant from a statistical point of view (p < 0.05).

As for kinetic parameters (ankle power), both PWS and DS showed lower maximum ankle power during terminal stance (APMax index; PWS: 1.96 ± 0.56 W/Kg; DS: 1.35 ± 0.64 W/Kg; p = 0.0003) than CG (3.07 ± 0.86 W/Kg), with DS significantly more limited than PWS. The APMax index normalised to the velocity of progression (APMax norm index) did not reveal significant differences among groups (PWS: 2.05 ± 0.49 m/s^2^; DS: 2.02 ± 0.86 m/s^2^; CG: 2.42 ± 3.06 m/s^2^; PWS vs. DS: p = 0.2261)

**Figure 1:**
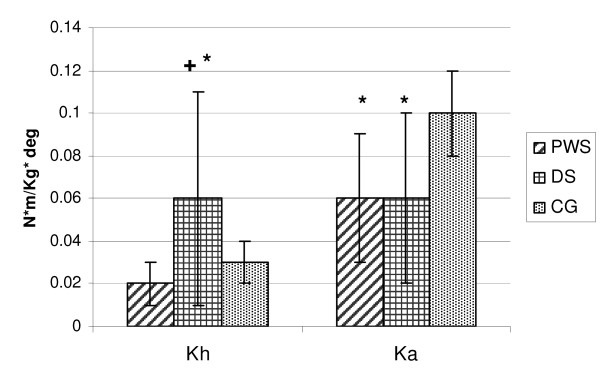
**Joint stiffness values of the study groups**. Data are expressed as mean (standard deviation). + = p < 0.05, PWS GROUP versus DS GROUP; * = p < 0.05 compared with Control Group. Kh: hip stiffness; Ka: ankle stiffness.

**Figure 2:**
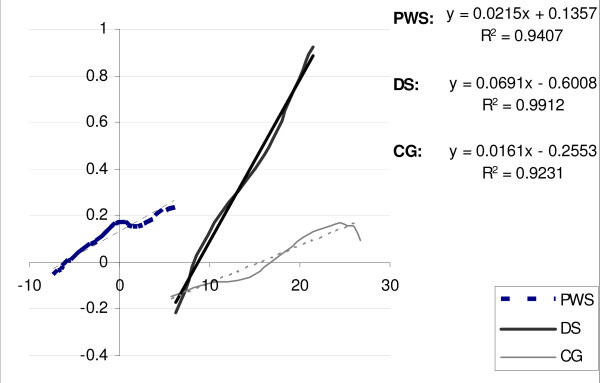
**An example of hip angle-moment plot cycle during second rocker for a participant with PWS, for one with DS and one healthy individual is reported**. The slope of the joint moment plotted as a function of joint angle during second rocker represents hip joint stiffness.

Joint stiffness data are shown in Fig. 1. PWS and DS were significantly different in terms of hip stiffness (Kh index): while PWS showed mean values close to CG, DS showed a significantly stiffer hip as compared to PWS (p < 0.0368) and CG. As for ankle stiffness (Ka index), no statistical differences were found between PWS and DS (p = 0.7716): both groups were characterised by reduced values as compared to CG. An example of hip stiffness for a PWS, a DS and a CG subject is shown in Fig. 2.

## Discussion

The aim of this comparative study was the quantification of spatio-temporal, kinematic and kinetic parameters during gait in patients affected by PWS and DS. While gait pattern in DS has been previously addressed, limited evidence exists in the PWS population. Lacking objective functional data, evidence-based rehabilitation strategies for PWS have failed to be implemented. From a clinical perspective, the biomechanical comparison of gait in these two genetic conditions sharing some clinical and functional features may provide a basis for developing either deficit-specific or common rehabilitative strategies.

The results of our study revealed that these two syndromes are characterised by different gait patterns. With regard to spatio-temporal parameters, both PWS and DS walk with longer stance duration, reduced anterior step length and lower velocity of progression when compared to CG. These parameters indicate a cautious, abnormal gait in both groups, aiming at balance and stability in individuals who bear an excessive body weight [18]. The comparison between PWS and DS outlined significant differences in terms of cadence, anterior step length and progression velocity. PWS were in fact characterised by values closer to normal than DS and are were able to walk with a more "stable" strategy.

Hip flexion was present throughout the gait cycle in PWS and DS, due to a forward pelvic tilt. PWS, however, were characterised by a more pronounced flexion at the initial contact than DS (HIC index). This strategy allows PWS a fair hip range of motion during gait (HFE-ROM index), whereas DS showed a limited excursion. The reason for that may be linked to the anatomical configuration of their pelvic girdle: the so-called "mongol pelvis" is characterised by a deeper acetabulum and a decrease in the cephalo-caudal diameter and acetabular angle [10,19].

In the frontal plane, hip excursion (HAA-ROM index) was higher in PWS than DS and CG. This strategy, directly linked to the pelvis movement in the frontal plane (PO-ROM index), appears to produce together with obesity and hypotonia the typical external rotation of the hip during stance [20]. This may account for faster walk and longer steps in PWS as compared to DS.

As for ankle kinematics, DS were characterised by an increased plantar flexion and reduced dorsal flexion throughout the gait cycle with a globally limited ankle range of motion. On the contrary, PWS showed an ankle strategy close to normal, apart from a slight plantar flexion at the initial contact and an increased dorsal flexion during swing. The PWS group was generally characterised by a wider, closer to normal range of motion in all of the lower limb joints in the sagittal plane.

In terms of ankle kinetics, PWS and even more DS showed lower peak ankle power than CG (APMax index), meaning a lower propulsion capacity during terminal stance. This result was consistent with previous studies [21]. Two possible hypotheses can be formulated for this limitation. Firstly, lower gait velocity in PWS and DS may affect ankle power. After normalising APMax index by gait velocity (APMax norm index) no significant differences among groups were evident. Secondly, the reduced push-off may be linked to muscle weakness which is a general feature of these patients. In particular, the triceps surae, mostly responsible for the generation of ankle power, may ineffectively contract during terminal stance. Capodaglio et al. [22] demonstrated that PWS patients have a reduced muscular strength as compared to weight-matched non genetically obese patients. Relative muscle weakness inducing earlier fatigue has also been described in obese patients [23].

Interestingly, we found differences in joint stiffness in PWS and DS. At hip level, PWS showed values close to normal, while in DS increased stiffness values were measured. At ankle level, joint stiffness was significantly decreased in both groups.

It is known that hypotonia and ligament laxity are common in PWS [24] and DS [25]. Our results suggest that the degree of hypotonia and ligament laxity may vary across various joints, being higher at ankle level where stiffness is decreased in both DS and PSW.

The increased hip stiffness in DS we found is consistent with the literature and may represent, together with the anatomical configuration of the pelvic girdle [26], a compensatory mechanism for muscle weakness [12]. Stiffness values closer to the normal range suggest a more "physiological" walking strategy in PWS than DS.

A potential weakness of this study may be the variability of data, since PWS and DS patients were evaluated in two different laboratories. However, we had previously compared markers' placement, procedures and data from normal-weight subjects in the two laboratories and no inconsistencies between laboratories occurred. Another bias of the study is that participants were not compared in terms of orthopaedic characteristics. PWS patients tend to develop a range of orthopaedic problems including scoliosis, hip dysplasia, flat feet, and pain syndromes of the lower limbs which may have an impact on gait. Also, the degree of muscular hypotonia and weakness, ligament laxity and cognitive impairment had not been measured nor compared between groups, thus hindering interpretation of the findings. As overweight is a distinctive feature in both PWS and DS, their gait pattern should have been more rigorously compared with obese instead of normal-weight individuals. However, the main object of our investigation was to compare gait strategy in PWS and DS patients to identify possibly common rehabilitation strategy.

## Conclusions

From a clinical point of view, quantitative characterisation of gait patterns in PWS and DS is important to develop, differentiate and enhance the rehabilitative options. The quantification of their peculiar gait deficits strongly support the issue that PWS and DS patients need targeted rehabilitation and exercise prescription. Common to PWS and DS is the aim to improve hypotonia, muscle strength and motor control during gait. Both patient groups should be encouraged to walk for its positive impact on muscle mass and strength and energy balance. In DS, improving pelvis and hip range of motion should represent a specific major goal to optimize gait pattern and prevent the onset of compensatory strategies. Evidence-based rehabilitation programs would contribute to improve daily functioning, quality of life and weight management issues in those patients.

## Competing interests

All authors haven't any conflicts of interest and any financial interest.

All authors attest and affirm that the material within has not been and will not be submitted for publication elsewhere

## Authors' contributions

VC made substantial contributions to analysis and interpretation of data and was involved in drafting the manuscript. MG made contribution to conception, design and interpretation of data, revising the manuscript critically and gave the final approval of the manuscript. GG made contribution to interpretation of data, revising the manuscript critically. LV made substantial contributions to data acquisition, elaboration and interpretation. GA made contribution to interpretation of data, revising the manuscript critically. CR made contribution to interpretation of data and to revision of the final version of the manuscript. PC made contribution to conception, design and interpretation of data, revising the manuscript critically and gave the final approval of the manuscript. All authors read and approved the final manuscript.
